# Metal Material, Properties and Design Methods of Porous Biomedical Scaffolds for Additive Manufacturing: A Review

**DOI:** 10.3389/fbioe.2021.641130

**Published:** 2021-03-26

**Authors:** Yuting Lv, Binghao Wang, Guohao Liu, Yujin Tang, Eryi Lu, Kegong Xie, Changgong Lan, Jia Liu, Zhenbo Qin, Liqiang Wang

**Affiliations:** ^1^College of Mechanical and Electronic Engineering, Shandong University of Science and Technology, Qingdao, China; ^2^State Key Laboratory of Metal Matrix Composites, Shanghai Jiao Tong University, Shanghai, China; ^3^Affiliated Hospital of Youjiang Medical University for Nationalities, Baise, China; ^4^Renji Hospital, Shanghai Jiao Tong University, Shanghai, China; ^5^Tianjin Key Laboratory of Composite and Functional Materials, School of Material Science and Engineering, Tianjin University, Tianjin, China

**Keywords:** metal material, additive manufacturing, porous scaffold, design, bone tissue engineering

## Abstract

Design an implant similar to the human bone is one of the critical problems in bone tissue engineering. Metal porous scaffolds have good prospects in bone tissue replacement due to their matching elastic modulus, better strength, and biocompatibility. However, traditional processing methods are challenging to fabricate scaffolds with a porous structure, limiting the development of porous scaffolds. With the advancement of additive manufacturing (AM) and computer-aided technologies, the development of porous metal scaffolds also ushers in unprecedented opportunities. In recent years, many new metal materials and innovative design methods are used to fabricate porous scaffolds with excellent mechanical properties and biocompatibility. This article reviews the research progress of porous metal scaffolds, and introduces the AM technologies used in porous metal scaffolds. Then the applications of different metal materials in bone scaffolds are summarized, and the advantages and limitations of various scaffold design methods are discussed. Finally, we look forward to the development prospects of AM in porous metal scaffolds.

## Introduction

Bone defects caused by pathologies such as fracture, bone tumor, or external trauma are among the main problems in clinical treatment (Moiduddin et al., 2017). Autologous bone transplantation is considered to be a good choice, but the mismatched performance of different bone sites and the limited number of useful bone grafts limit the application of autologous bone transplantation (Henkel et al., 2013). In contrast, allogeneic bone transplantation has an obvious risk of immune rejection and infection, which affects bone formation and is prone to bone resorption. Therefore, it is ideal to seek natural bone replacement for bone transplantation in orthopedics.

As an alternative material, porous metal scaffolds avoid a series of adverse reactions in natural bone grafting and have gradually attracted researchers’ attention. To simulate the mechanical properties and biocompatibility of real bone, porous metal scaffolds not only have interconnected porous structures but also have good mechanical properties and biocompatibility (Li et al., 2020a). Mechanical properties mainly include better yield strength, matching elastic modulus, and better fatigue strength (Yuan et al., 2019). Common biomedical metal materials such as Ti and Ti alloys can completely meet bone implants needs in terms of strength. Nevertheless, the elastic modulus of dense metals is much greater than that of human bones, which is prone to bone resorption and leads to bone loosening in the human body (Bundy, 2008). The porous scaffolds can obtain matching elastic modulus with human bone by adjusting the pore size and porosity (Kelly et al., 2019), and at the same time have better yield and fatigue strength (Chen et al., 2018). Porous metal scaffolds should also have good biocompatibility, which not only can promote cell attachment, growth, proliferation, and differentiation, but also facilitates the transport of nutrients and metabolic wastes (Little et al., 2011; Saint-Pastou Terrier and Gasque, 2017).

Traditional processing methods are challenging to prepare porous metal scaffolds with complex structures, while additive manufacturing (AM) technology can prepare the scaffolds with controllable structures, shape, and properties (Wang et al., 2020a). Thus AM is one of the most effective methods to prepare porous metal scaffolds. The design of porous metal scaffolds is another crucial problem because scaffold features such as unit type, pore size, porosity, and distribution have significantly influence on their mechanical properties and biocompatibility. Therefore, this article introduces the AM technologies for preparing metal scaffolds and summaries the research progress in relative metal materials, including non-biodegradable metals (Ti alloys, Ta alloy, and stainless steel), and biodegradable metals (Fe, Mg alloy, and Zn alloy). Besides, we review the structural characteristics of porous metal scaffolds and their design methods in detail, and evaluate the advantages and limitations of these methods. Finally, we prospect the future development direction of bone scaffolds.

## Basic Requirements for Metal Porous Scaffolds

For metal implants, the elastic modulus is a very important mechanical performance (Ngo et al., 2018). Large elastic modulus differences between the implants and the bone tissue can result in “stress shielding” effect, which will gradually trigger the loosening of the implant, finally leading to the failure of implant. As known to all, solid metals has much higher elastic modulus than bone tissue (Li et al., 2020a). Obviously, the solid metals are not suitable to use as implants. Thus porous structures were designed in order to reduce the elastic modulus of the solid metals. Metal porous implants should be non-toxic, non-rejection, and non-allergenic, which requires us to select suitable metal as raw material (Roseti et al., 2017). Good biocompatibility is also reflected in the reasonable porous shape and distribution, which can promote the adhesion and growth of bone tissue cells (Shor et al., 2007). In addition, metal porous scaffolds should have good wear and corrosion resistance. Worse wear resistance can cause loosening of the scaffolds, and metal particles caused by wear or metal ions formed due to the corrosion effect can lead to tissue reactions and lesions (Wang et al., 2020a). Furthermore, the scaffolds should have good machinability, and the structures can be obtained using existing processing technologies.

## Additive Manufacturing Technology

Additive manufacturing (AM) technologies, also known as 3D printing, attracts extensive attention in the fabrication of biomedical implants due to their capability of manufacturing porous scaffolds with irregular shapes (Chen et al., 2020b). AM prepares products by layer-by-layer stacking method, which divides into the following three steps. Firstly, the entity model is established by commercial software such as UG, Pro/Engineer, SolidWorks, and Materialise 3-Matic, etc. Secondly, the model is imported into slicing software for slicing and layering. Finally, the layered file is imported into a 3D printer, and the parts are formed layer by layer from bottom to top. At present, the AM technologies suitable for preparing porous metal scaffolds mainly divides into two categories: powder bed fusion technology (PBF) and directional deposition technology (DED) (Chen et al., 2020a). Compared with DED, PBF can prepare the parts with better manufacturing accuracy and surface quality and are more prevalent in the biomedical field. Therefore, this article focuses on powder bed fusion technologies, including selective laser sintering (SLS), selective laser melting (SLM), and electron beam melting (EBM) (Chen et al., 2020b). The differences in these AM technologies are summarized, as shown in Table 1.

**TABLE 1:** Summaries of four different additive manufacturing technologies: selective laser sintering (SLS), selective laser melting (SLM), electron beam melting (EBM), and directional deposition technology (DED).

Category	Materials	Application	Resolution (μm)	Advantages	Disadvantages	References
SLS	Polymers Metals Alloys	• Biomedical fabrication • Shipbuilding • Auto industry • Aerospace	76–100	• Superior mechanical properties • Complex geometry • No supporting • High utilization of powder Materials	• Low energy efficiency • Expensive Low density	Chohan et al., 2017; Ngo et al., 2018
SLM	Metals Alloys	• Biomedical fabrication • Shipbuilding • Auto industry • Aerospace	80–250	• Superior mechanical properties • Complex geometry • No supporting • High density	Expensive • Residual stress • Rough surface • Time consuming process	Sood et al., 2010; Tofail et al., 2018
EBM	Metals Alloys	• Biomedical fabrication • Shipbuilding • Auto industry • Aerospace	50–100	• Superior mechanical properties • Complex geometry • No supporting	Expensive • Rough surface • Time consuming process	Ngo et al., 2018; Qu, 2020
DED	Metals Alloys Ceramics Glass Polymers	• Aerospace • Repair of bespoke parts • Biomedical application	250	Good mechanical properties • Rapid cooling rates • Effective time and cost of repairs	Low resolution • Low surface quality • Producing less complex • parts	Gibson et al., 2015; Mohamed et al., 2015

### Selective Laser Sintering (SLS) and Selective Laser Melting (SLM)

**FIGURE 1:**
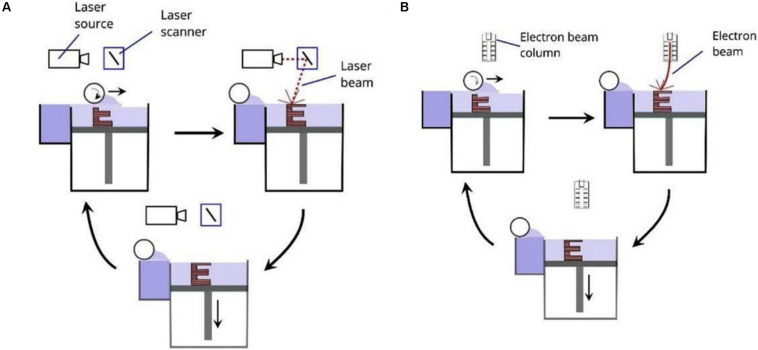
Schematic diagrams of PBF including **(A)** SLS and SLM, and **(B)** EBM (Ataee et al., 2017).

Figure 1A show the schematic diagram of SLS. SLS uses a laser as an energy source to sinter the powder materials (Szymczyk-Ziółkowska et al., 2020). After melting one layer, the equipment descends to fabrication platform and raises the powder delivery platform. Then the roller rolls out powders on the fabrication platform, and a new layer of sintering begins. This process is repeated until entirely formation of the part. When using SLS, prepared material need introduce binder materials (alloys with a low melting point) to reduce the melting point, promoting sintering (Lee et al., 2017). SLS can prepare a variety of materials such as polymers (Goodridge et al., 2012), metals and alloys (Bae et al., 2014), etc. but it is challenging to prepare metal materials with a high melting points.

Selective laser melting is developed based on SLS technology, and its principles are the same. Nevertheless, powder material and the bonding mechanisms in the two technologies are different. In SLS technology, the powder materials are heated to partly melt by laser beams instead of completely melting (Bose et al., 2018). Powders with a low melting points are used as binders for bonding high melting point metals (Qu, 2020). Compared with SLS, the laser of SLM has higher energy (Dogan et al., 2020), which can completely melt the powder. Thus it can prepare metals or alloys with a high melting points. The parts prepared by SLM have higher dimensional accuracy and density, and their mechanical properties are comparable to those of forged one. Due to the high sintering temperature, the powder sintering needs to be performed under the protection of inert gas to prevent metal oxidation (Wang et al., 2017).

Selective laser melting technology also has shortcomings. The surface of parts prepared by SLM can adhere to some particles that do not melt completely (Zadpoor, 2019), resulting in its high surface roughness. It is necessary to smooth the surface by sandblasting or chemical corrosion (Ahmadi et al., 2019; Zadpoor, 2019). Besides, when the SLM is used to process brittle materials, residual stresses are easily generated inside the parts during the cooling process. Thus it is often necessary to adopt isobaric sintering or heat treatment to eliminate residual stress (Fang et al., 2020).

### Electron Beam Melting (EBM)

Electron beam melting, like SLS and SLM, is a powder bed fusion technology (PBF). The significant differences between SLM and EBM are the source of energy, and their energy sources are laser and high-energy, high-speed electron beams, respectively. Figure 1B show the principle of EBM. The electron gun emits electrons, and then the electron beam is accelerated by the heated tungsten wire, and the direction and diameter of the electron beam are controlled by a magnetic lens (or coil) (Yuan et al., 2019). During sintering, the metal power melts entirely, and then the electron beam sweeps the powder along the preset path and processing parameters. The powders can be melted and solidified into a metal entity, finally forming a thin layer with a thickness of 0.05–0.2 mm. Then the powders are spread and sintered again and this operation was repeated until the formation of parts entirely. To prevent the oxidation of the metal powder, the entire process needs to be carried out in a vacuum (Gokuldoss et al., 2017). EBM’s advantages are lies in the higher energy density of the electron beam, faster rate of powder melting and forming (Liu et al., 2016). Due to the high energy density, EBM technology can prepare refractory metals with high melting points. During processing, EBM usually heats the powder bed, which can reduce the temperature differences between the powder bed and the metal. Thus the residual stress of parts is small (Gokuldoss et al., 2017). Like the SLM technology, the parts prepared by EBM have high surface roughness (Gong et al., 2014), and the dimensional error and surface integrity are worse than those of the cast one.

## The Metal Material Used in Porous Scaffolds

### Non-biodegradable Metals

The non-biodegradable metal materials currently used in porous scaffolds mainly include pure Ti (Wauthle et al., 2015a), Ti alloy (Kapat et al., 2017; Onal et al., 2018; Cutolo et al., 2020), Ta (Wauthle et al., 2015b), 316L stainless steel (Yan et al., 2014), NiTi alloy (Habijan et al., 2013), and Co-Cr alloy (Demir and Previtali, 2017), etc. Table 2 lists the mechanical properties of different non-biodegradable porous scaffolds (Peng et al., 2019). Ti alloys are widely used in orthopedic implants due to the better biocompatibility, corrosion resistance, and excellent mechanical properties (Zhu et al., 2016). Ti-6Al-4V has more matching elastic modulus with human bone and relatively low price (Yang et al., 2018; Lv et al., 2019), which is the most studied biomedical Ti alloy (Cheng et al., 2014; Zhu et al., 2018). However, cytotoxicity experiments of Ti-6Al-4V scaffolds also indicate that the release of Al and V ions occurs in the human body, which affects cell proliferation and causes cytotoxicity (Surmeneva et al., 2019). Pure Ti, as a good biometal material, avoids the release of harmful ions (Liu J. et al., 2020). Wauthle et al. (2015b) and Liu S. et al. (2020) prepared pure Ti scaffolds with a dodecahedral unit structure and found that the scaffolds have higher fatigue cycle strength and ductility than that of Ti-6Al-4V. The Nb and Zr elements also have good biocompatibility (Sing et al., 2016) and have been used as alloying elements to improve the biological and mechanical properties of Ti alloys (Lin et al., 2013). Liang et al. (2020) prepared Ti-25Nb porous scaffolds with a hydrophilic surface structure. They found that Ti-25Nb scaffold can promote the expression of phagocyte M2 type and enhance the activity of anti-inflammatory phagocytes. Luo et al. (2020) found that the Ti-30Nb-5Ta-8Zr scaffold exhibits similar fatigue strength, compression, and tensile properties with cortical bone, and they also established the functional relationship between the porosity, yield strength, and elastic modulus of the alloy. Wauthle et al. (2015b) and Wang H. et al. (2019) prepared three type porous metal scaffolds, including Ta, pure Ti, and Ti-6Al-4V. They found that Ta porous scaffolds have the same cell proliferation, survival and osteogenic properties as Ti scaffolds. Moreover, the Ta scaffolds have better toughness and fatigue limit than Ti-6Al-4V scaffolds (Guo et al., 2013). However, Ta scaffold has a higher price, which limits its wide application.

**TABLE 2:** Mechanical properties of different porous metal scaffolds.

Mechanical properties of porous metal scaffolds			

Materials(structure)	Elastic modulus (GPa)	Yield strength (MPa)	References
Ti-6Al-4V (Gyroid and Diamond)	3.8	152.6 145.7	Liu et al., 2018
Ti-6Al-4V (Octahedral)	2.1–4.7	71–190	Yan et al., 2019
Pure Ti (Diamond)	0.557–0.661	50	Taniguchi et al., 2016
Pure Ti (FGPS)	0.28–0.59	3.79–17.75	Han et al., 2018
Pure Ta (Diamond)	3.1	393.62	Wang H. et al., 2019
Pure Ta (Dodecahedron)	1.22	12.7	Wauthle et al., 2015b
Ti-30Nb-5Ta-8Zr (Rhombic dodecahedron, Body diagonals)	0.7–4.4	12.5–67	Luo et al., 2020
Ti35Zr28Nb (Face centered cubic)	1.1	27	Li et al., 2019a
Ti-35Nb-2Ta-3Zr	3.1 3.5 3.9	136 137 149	Hafeez et al., 2020
CoCr F75 (Diamond)	3.43 2.32 2.22	116.34 75.97 78.57	Hooreweder et al., 2017
NiTi (Octahedron, Cellular gyroid, Sheet gyroid)		21 29 44	Speirs et al., 2017
NiTi	3.7–13.5		Bartolomeu et al., 2020; Liu S. et al., 2020
316L (Gyroid)	2.04 2.48 2.71	55 72.1 89.4	Ma et al., 2019
316L (Gyroid)	14.41–15.53	251–302	Yan et al., 2014
Fe (Diamond)	2.81 0.89 1.77 1.75	53.1 10.7 32.9 30.5	Li et al., 2019b
Fe-35Mn (Schwarz Primitive)	33.5	304	Carluccio et al., 2020
Zn (Diamond)	0.786	10.8	Li et al., 2020c
Mg WE43 (Diamond)	0.7–0.8	23	Li et al., 2018

Ma et al. (2019) prepared 316L stainless steel porous scaffolds and studied the influence of the pore size and porosity on their elastic modulus, yield strength, and permeability. They also established the functional relationship of the above parameters and predicted the permeability of the scaffold. Čapek et al. (2016) also obtained 316L stainless steel scaffolds with good mechanical properties and found that the mechanical properties are close to that of trabecular bone. However, compared with Ti alloy and Ta, stainless steel has a higher elastic modulus, which easily leads to stress shielding (Yamamoto et al., 2004). Thus how to adjust the pore size and porosity of scaffolds and balance their relationship between strength and elastic modulus is the crucial point for 316L stainless steel porous scaffolds.

NiTi alloy has the characteristics of superelasticity and shape memory, which have a good application prospect in biomedical field (Wang L. et al., 2016; Liu et al., 2019). Nevertheless, the Ni ions in the alloy have cytotoxicity, which is a concerning matter to people. Habijan et al. (2013) prepared NiTi scaffolds with different porosity and surface morphology, and cultivated stem human mesenchymal stem cells (hMSC) on NiTi scaffolds. They found that the amount of Ni released in the porous scaffold is higher than that of the dense sample, but all were below the cytotoxic concentration. They also found that changing the spot diameter can improve the scaffold surface morphology, and reducing the spot diameter can reduce Ni ions’ release. They believed that NiTi scaffolds are suitable carriers for hMSC, but the process parameters and post-processing still need to be optimized before *in vivo* studies (Liu et al., 2021).

The Co–Cr alloy has good biocompatibility (Baldwin and Hunt, 2006), corrosion resistance, and wear resistance, and is widely used in orthopedic surgery, especially in hip replacement or knee replacement. However, the osseointegration and biomechanical properties of Co–Cr alloy are inferior to Ti-6Al-4V. Shah et al. (2016) prepared Co–Cr and Ti-6Al-4V porous scaffolds by EBM. *In vivo* implantation experiments found that the bone-implant bonding rate of the Co–Cr scaffold is lower than that of the Ti-6Al-4V, but they have similar bone cell density and distribution in a newly formed bone. Caravaggi et al. (2019) prepared Co–Cr scaffolds with different porous structures by SLM, and found that the elastic modulus of porous structure is about 32 GPa, which is close to the elastic modulus of human bone. Cell culture experiments showed that the number of cells on the porous structure continued to increase over the course of 1 week, indicating that the Co–Cr alloy had good biocompatibility.

### Biodegradable Metals

The biodegradable metal can effectively avoid chronic local inflammation (Moravej and Mantovani, 2011), continuous physical stimulation (Song and Song, 2007), and implant-related infections, which has broad prospects in the biomedical field. At present, Fe, Mg, and Zn alloys are widely studied as materials for degradable scaffolds (Li et al., 2020b). How to match the rate of metal degradation to that of bone tissue ingrowth is the main challenge. Table 2 lists the mechanical properties of Fe, Mg, and Zn porous scaffolds.

**FIGURE 2:**
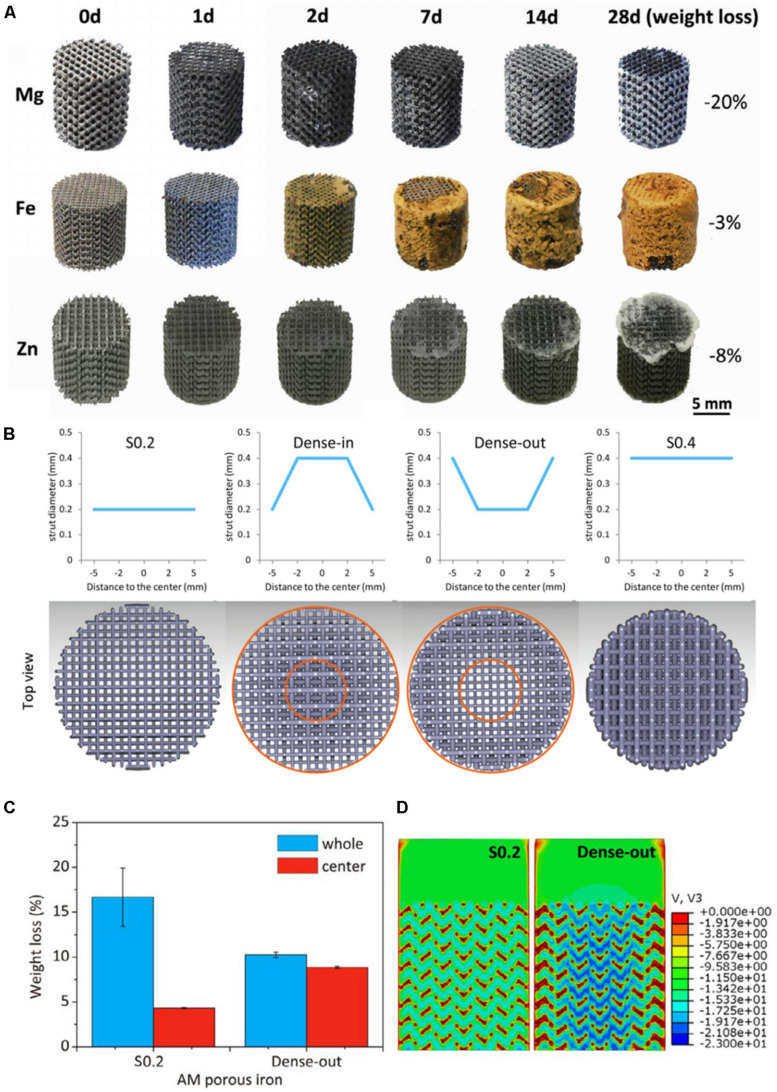
**(A)** The morphologies of samples after *in vitro* immersion tests (Li et al., 2020b). **(B)** Functionally graded structure of porous Fe scaffolds. **(C)** The weight of S0.2 and Dense-out for 28 days. **(D)** The flow distributions in S0.2 and Dense-out according to CFD modeling (Li et al., 2019b).

Fe is an element needed by the human body, and also has good biocompatibility. The main problem for Fe is the slower degradation rate in the human body, which can inhibit the ingrowth rate of bone tissue. Li et al. (2019b) prepared gradient porous Fe scaffolds and the pore size of scaffolds are 600 μm (S0.4), 600–800 μm (Dense-out), 800–600 μm (Dense-in), and 800–600 μm (S0.2). They found that the scaffold of S0.2 and Dense-out had exactly the same structure in the center (Figure 2B), but the weight loss of the Dense-out scaffold in the center was higher than that of the S0.2 scaffold, as shown in Figure 2C. They believe that the Dense-out scaffold has higher flow velocities in the center than on the periphery, as shown in Figure 2D. Adding alloy elements into the Fe can also affect its degradation rate. Carluccio et al. (2019) prepared Fe and Fe–Mn porous scaffolds. They found that the corrosion rate of Fe–Mn scaffold is much higher than that of pure Fe. They believed that a galvanic cell is formed between the different metal scaffolds, which accelerates Fe–Mn alloy’s degradation. The Fe–Mn alloy scaffold has good biocompatibility and vitality to mammalian cells.

Mg alloy porous scaffolds have a higher degradation rate, leading to its complete degradation before bone tissue fully grows into the scaffolds. To decrease the degradation rate, surface modification (plasma electrolytic oxidation), and heat treatment of the scaffold were performed by Kopp et al. (2019). They found that Mg hydroxide and oxide are formed on the scaffold surface, which slows down the degradation rate in the simulated body fluid. Mg is more active, and there are problems such as difficulty in preparation, powder splashing, cracks, and so on (Wang et al., 2020d). The degradation rate of Mg scaffolds also can produce hydrogen that affects cell proliferation. They believed that if the problems mentioned above can be dealt with, Mg alloys will have a more significant impact in the biomedical field.

Zn alloys have gradually attracted extensive attention from researchers because their degradation rate is closest to bone tissue (Su et al., 2019; Fu et al., 2020), which is very beneficial to the healing of bone tissue. Li et al. (2020c) prepared Zn scaffolds with a diamond structure and found that the mechanical properties are similar to cancellous bone. The volume loss is 7.8 and 3.6%, respectively, after 28 days of dynamic and static immersion *in vitro*, and the degradation rate is between Mg and Fe, as shown in Figure 2A. The mechanical properties of the Zn scaffolds after soaking can be improved after a small amount of degradation. Cockerill et al. (2020) prepared Zn scaffolds with different pore sizes through combination methods of AM and casting and found that the Zn scaffolds have good biocompatibility and antibacterial properties.

### High Entropy Alloys

Compared with traditional metals and alloys, high-entropy alloys are gradually becoming a focus of attention due to their better comprehensive properties. These alloys are no longer based on a particular component, but are made of a variety of metal to provide better properties such as strength, corrosion resistance, and biocompatibility (Ma et al., 2020).

Motallebzadeh et al. (2019) prepared TiZrTaHfNb and Ti_1.5_ZrTa_0.5_Hf_0.5_N_*b0.5*_ high entropy alloys and compared their properties with 316L, CoCrMo, and Ti6Al4V alloys. They found that the high entropy alloy show higher wear resistance and corrosion resistance. They attributed the higher mechanical properties to the “cocktail effect” of the high entropy alloy. Nagase et al. (2019) developed novel TiZrHfCr0_.2_Mo and TiZrHfCo_0.07_Cr_0.07_Mo high-entropy alloys for metallic biomaterials based on the combination of Ti–Nb–Ta–Zr–Mo and Co–Cr–Mo alloy systems. The experimental results showed that newly developed high entropy has comparable biocompatibility with pure Ti.

## The Structure Design of Porous Metal Scaffolds

The ideal scaffold should be a porous structure in space that provides space for cells to adhere, grow and proliferate, and have mechanical properties similar to the bone tissue (Cheng et al., 2014). Pore size and porosity are very important structural parameters, which have a direct impact on mechanical properties and biocompatibility of bone scaffolds. Proper pore size can provide growth space for cells, and proper porosity can ensure transportation of nutrients and metabolites in bone tissue (Cheng et al., 2014). Besides, the shape of the porous scaffold structure is also related to the biocompatibility and mechanical properties. The continuous and smooth porous structure can avoid stress concentration and facilitate the attachment of cells to the scaffold surface.

Porous scaffold prepared by the traditional foaming (Murray, 2003) and sintered microsphere methods (Mark et al., 2002) has a single structural unit, and the shape, size, and spatial distribution can not be precisely controlled. With the development of computer-aided design and AM technologies, problems as mentioned above have gradually been improved. AM technologies not only can accurately control the porous scaffold size and spatial structure distribution but also can obtain ideal mechanical properties and biocompatibility of porous scaffold by adjusting the pore size and porosity. In this section, the pore size and porosity of the scaffold are described, and the influence of pore size and porosity on the scaffold’s performance are summarized. Then current design methods for porous metal scaffold including CAD structure, topology optimization, minimal surface structure, Voronoi mosaic method, CT imaging method, etc. are systematically reviewed.

### Pore Size and Porosity of the Porous Metal Scaffold

**FIGURE 3:**
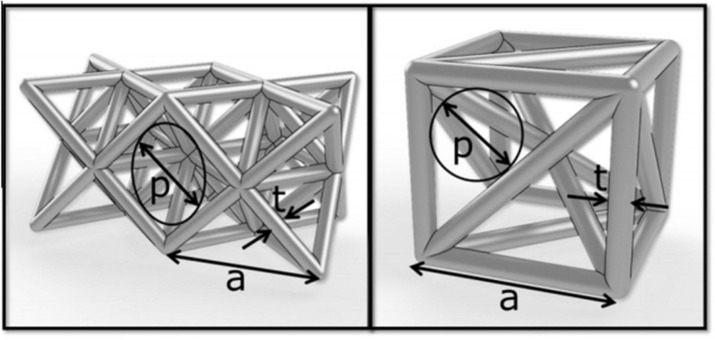
Basic structural unit: p: aperture, t: pillar thickness (Arabnejad et al., 2016).

The porous scaffold’s pore size is generally defined by the inscribed circle method, as shown in Figure 3. The definition of porosity is the percentage of pore space in the solid structure given by the following formula:

Porosity = (1-*V*_*P*_/*V*_*S*_) × 100%

Among them: *V*_*P*_is the volume of the porous structure and *V*_*S*_ is the volume of the dense structure.

#### The Influence of Pore Size and Porosity on Biocompatibility

Many investigations reported that the optimal pore size and porosity of the porous scaffold are about 200–1200 μm (Ataee et al., 2018) and 60–95% (Zhou et al., 2015), respectively. To explore a more specific range of porosity, Ma et al. (2020b) prepared convolute structured scaffolds with a porosity of 75–88% to study the effect of different porosity on cell proliferation. They found that a scaffold with a porosity of 88.8% has the largest number of cells, and they believed that increasing porosity could increase the specific surface area of the porous scaffold and improve its permeability.

Ouyang et al. (2019) explored the pore size’s effect on the biocompatibility of porous scaffolds and designed Ti-6Al-4V scaffolds with pore sizes of 400, 650, 850, and 1100 μm, respectively. They found that increasing pore size can reduce the thickness and specific surface area of the pillar and increase the scaffolds’s permeability. Cell proliferation and *in vivo* bone formation first increase and then decrease with the increase of pore size, and the best pore size is 650 μm. Ran et al. (2018) compared the bone ingrowth of Ti-6Al-4V scaffolds with a different pore sizes (400, 600, and 800 μm). They found that the bone ingrowth properties of the scaffold with pores size of 800 μm and 600 μm is significantly better than that of 400 μm.

Wang S. et al. (2019) compared the biocompatibility of Ti-6Al-4V scaffold with different structures, including OTC, TC, and OTC+TC (PFGS) structures. They found that the OTC structure has the fastest cell proliferation in 1–4 days, and the PFGS structure has the fastest cell proliferation in 4–7 days. In contrast, TC structure has slowest cell proliferation in 1–7 days. They believed that increasing the pore size can improve the permeability of the structure and high permeability can transport more oxygen and nutrients, which is conducive to cell growth in the early stage of cell culture (Wang et al., 2020c). PFGS has a smaller inner hole that is conducive to cell adhesion and differentiation, so the PFGS structure shows a higher cell proliferation rate in the later stage of cell culture.

#### The Influence of Pore Size and Porosity on Mechanical Properties

The dense metal materials have a much higher elastic modulus than human bone. For example, the elastic modulus of pure Ti and Ti-6Al-4V are 112 and 132 Gpa, respectively (Sing et al., 2016). While the elastic modulus of trabecular or cancellous bone is between 0.02 and 2 Gpa (Wang X. et al., 2016), cortical bone is higher, ranging from 7.7 to 21.8 Gpa (Zhang et al., 2018). At present, the implant’s elastic modulus is mainly controlled by adjusting the pore size and porosity. Yan et al. (2015) prepared Ti-6Al-4V scaffolds with G and D structures with a porosity of 80–95%, a pore size of 480–1600 μm, and found that the elastic modulus is about 0.12–1.25 GPa. The pore size and porosity of scaffold also have immediate impact on strength (Ran et al., 2018). Zhao et al. (2019) prepared octahedral structured Ti-6Al-4V porous scaffolds with pore sizes of 500 and 1000 μm and found that increasing pore size can decrease the tensile strength and fatigue strength. Therefore, increasing the pore size and can reduces the elastic modulus, but it will also cause a decrease in the tensile strength and fatigue strength of the scaffold.

### Structure Design Methods of Porous Metal Scaffold

#### CAD Method

**FIGURE 4:**
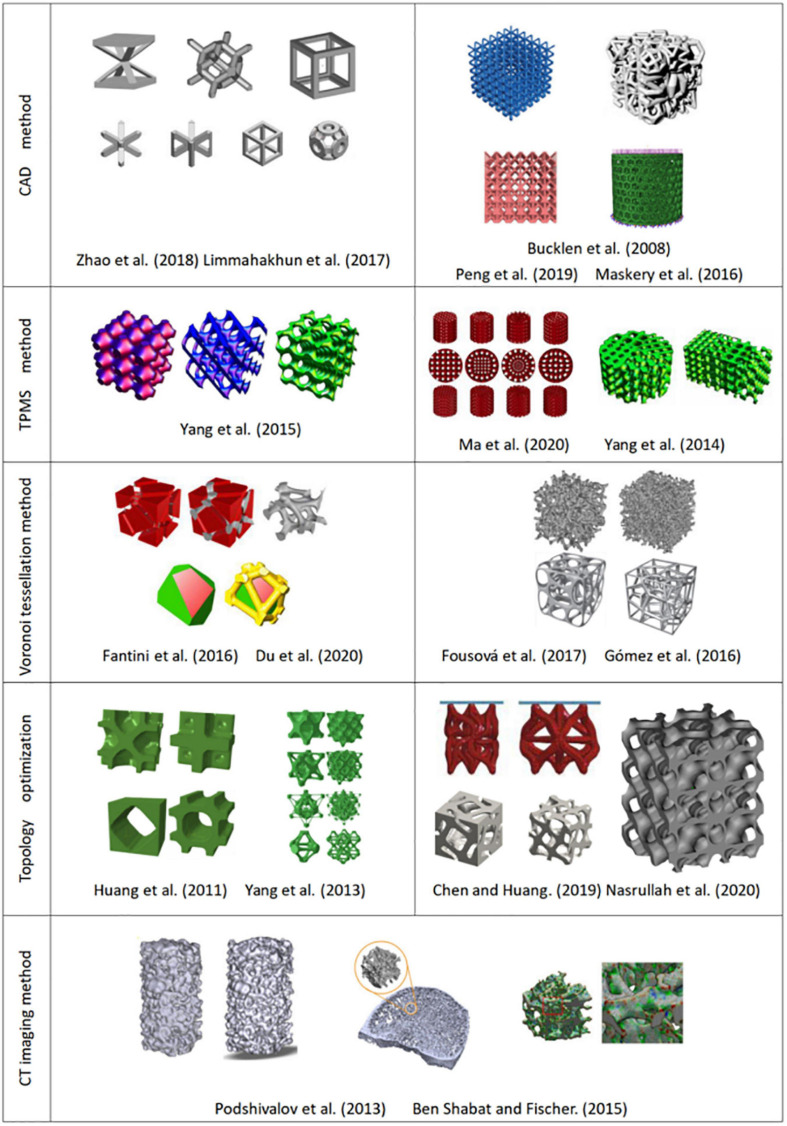
Structures designed by different design methods.

The main principle of the CAD method is to design different types of hole-making units and then create a porous scaffold through the Boolean operation (Zhao et al., 2018). As the basic unit of a porous scaffold, the shape, porosity, pore size, and surface area have a direct impact on the overall performance. Thus in the early stage, researchers mainly focused on the design of the hole-making unit. Based on bionic characteristics of different parts of human bones, Sun et al. (2005) designed structural units with disk and rod shapes using computer-aided tissue technology (CATE). They obtained a combination of different units by adding the same circular boundary on different units. Finally, a unit library that can combine multiple structural units was established. Chua et al. (2003) used the CAD method to develop a standard unit library containing 11 kinds of hole elements and developed an automatic assembly unit to match the anatomical shape of bone tissue. Polyhedra and lattice structure play an important role in CAD design due to their simple structure and good mechanical properties. Maskery et al., 2016 designed a gradient BCC (body centered cubic) lattice model (see Figure 4) to compare their mechanical properties and energy absorption with a uniform one. They found that the gradient structure is able to absorb around 114% higher energy than uniform structure. Li et al. (2019a) established the FCC (face-centered cubic structure) and BCC lattice models, and they considered that this simple and reliable models can obtain the desired mechanical properties and biocompatibility. Limmahakhun et al. (2017) established four structures (octahedron, column octahedron, cube and truncated octahedron), as shown in Figure 4. The mechanical tests and *in vitro* cellular experiments showed that the column octahedron structure can balance mechanical and biological properties, and are more suitable as the basic unit of bone scaffold. Although created units using CAD method are simple and have a low mechanical property (Wettergreen et al., 2005; Chantarapanich et al., 2012), the method still provides ideas for the following research.

#### Triple Periodic Minimal Surface (TPMS) Structure

A minimal surface is an implicit equivalent surface with zero mean curvature. If the minimal surface is periodic in three independent directions, it is usually called triple periodic minimal surface (TPMS). TPMS can be expressed by a trigonometric function, as shown in Table 3. Changing the TPMS’s threshold value can accurately control the internal pore structure, optimize the gradient pore structure, and maximize the specific surface area of the scaffold.

**TABLE 3:** Common minimal surface structures.

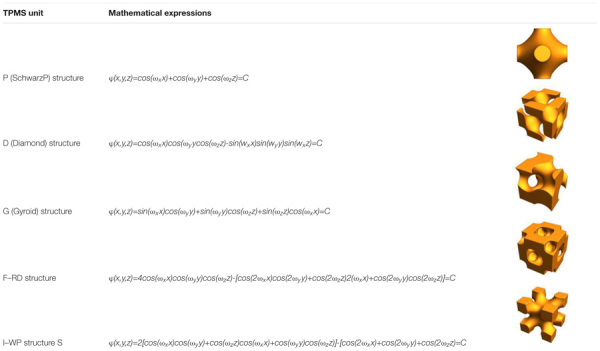

Yoo (2011b) proposed a three-dimensional bone scaffold design method that integrated distance field algorithms and TPMS curved surfaces. This method can automatically obtain a bone scaffold model with complex microstructures and high quality free-form external surfaces. Yoo (2011a) proposed another TPMS design method that two different TPMS structures can be combined by using a linear interpolation algorithm. Yang et al. (2014) reported that smooth transition between multiple different TPMS substructures could be combined by sigmoid and Gaussian radial basis functions (Yang and Zhou, 2014; Yang et al., 2015). Ma et al. (2020a) proposed a new method for designing heterogeneous porous scaffolds, that is, TPMS units were combined with grid units using shape functions, and they obtained a conformal refined discrete scaffold of a full hexahedral grid. After finite element analysis, they found that the elastic modulus, strength, and energy absorption of the heterogeneous scaffolds are significantly improved than uniform structure.

Nature bone has a porous gradient structure; thus, the gradient TPMS structure is a hot spot in scaffolds design (Almeida and Bártolo, 2014; Zhou et al., 2020). Wang et al. (2020c) designed a symmetrical gradient Ti-6Al-4V scaffolds with a P structure. They found that the gradient structure has better mechanical performance than that of the uniform structure. Zhang X.Y. et al. (2020) proposed a new method that flexible control of structural parameters can be realized by changing the TPMS equation and found that those structure design parameters have obviously effects on the scaffold performance.

#### Voronoi Tessellation Method

Voronoi tessellation is a space division method based on seed points (Du et al., 2020). The seed points are connected through a specific algorithm to form a space polygon surrounding the seed points. Based on these polygonal edges, irregular porous scaffolds are generated (Xiao and Yin, 2016). Thus the internal structure of natural bone can be well simulated by irregular porous scaffolds based on the Voronoi tessellation principle.

Kou and Tan (2010) proposed a design method with controllable shape and distribution by using the two-dimensional Voronoi diagram and they obtained irregular concave and convex polygons through the merging of Voronoi units. Then the boundaries of the concave and convex polygons were interconnected to form a bracket. The method makes a heterogeneous porous structure easier and maintain the irregularities in natural bone. Fantini et al. (2016) and Fantini and Curto (2017) used the three-dimensional Voronoi tessellation method to design porous structures, and obtained the three-dimensional Voronoi unit by processing the three-dimensional coordinates, and established the porous structure by Boolean operation on Voronoi unit, as shown in Figure 4. Lei et al. (2020) proposed a new Voronoi tessellation method to control the distribution of seed points and established a function relationship of the porosity and the number of seed points. Through this method, they obtained a Voronoi tessellation scaffold with a gradient distribution of seed points, which realizes the global control of the lattice porous structure.

The Voronoi tessellation method can generate an irregular pore model with controllable pore size and distribution, and the automation degree of generating the model is relatively higher. However, this method can not generate complicate porous structure due to the difficulty in the visualization of the porous scaffolds (Wang et al., 2020b).

#### Topology Optimization

Topology optimization technology is a mathematical method based on finite elements (Marinela et al., 2019), which can rearrange materials or structures to obtain the required mechanical properties (Zhao et al., 2014). It is a powerful method for the design of complex structures with multi-scale features (Chen and Huang, 2019).

Yang et al. (2013) proposed a topology optimization method of periodic hole unit structure and designed a porous scaffold with a required Young’s modulus, as shown in Figure 4. Radman et al. (2012) specified the volume or shear modulus of units, and optimized the primary unit through the anti-homogeneous two-way advanced optimization technology, and established functionally gradient porous structure by the proper connection between adjacent basic units. Xiao et al. (2012) rearranged the structure of the model under the constraint of volume fraction to achieve the ideal stiffness through the topology optimization method and obtain optimal three-dimensional structure of porous scaffolds (Xiao et al., 2013). Nasrullah et al. (2020) established 11 kinds of porous structures by topology optimization of lattice structures, and reported a conical lattice structure that can provide energy absorption of up to 127 kJ/kg. Zhang L. et al. (2020) combined the topology optimization with numerical homogenization method to design high stiffness lattice structure, and successfully obtained a new lattice structure with high load-bearing and energy absorption capacity, and the relative elastic modulus can reach 0.037.

Topology optimization methods can combine with a variety of design methods to achieve required mechanical properties and biocompatibility (Wang X. et al., 2016; Park et al., 2018). Nevertheless, the design methods have many variables and high calculations (Zhang X.Y. et al., 2019). How to balance the relationship between structural design and calculation efficiency remains to be resolved (Dias et al., 2014).

#### CT Imaging Method

**FIGURE 5:**
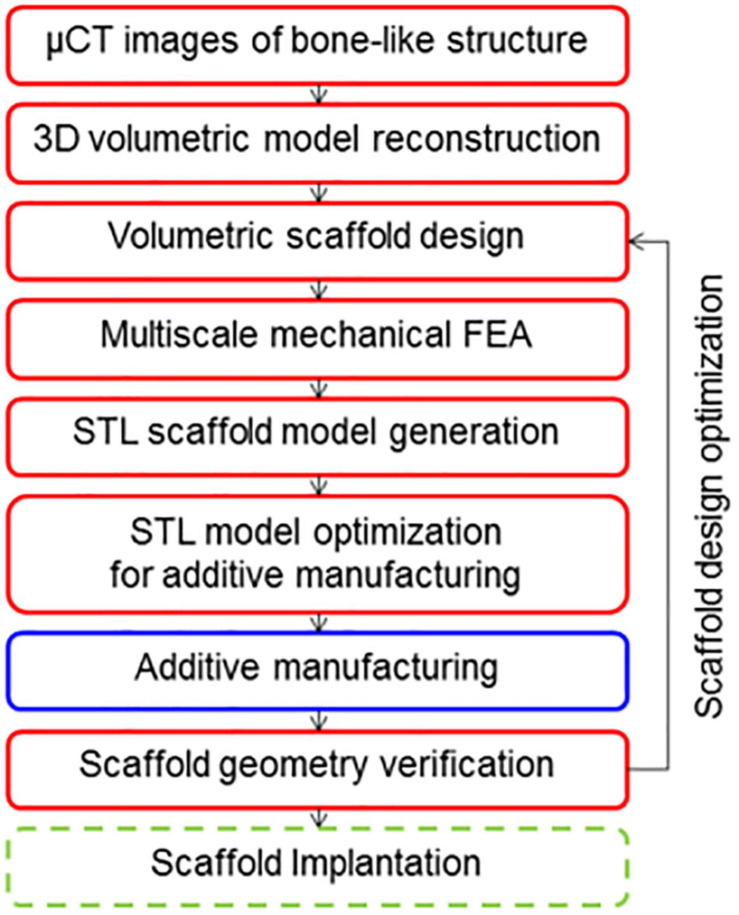
CT image modeling process (Podshivalov et al., 2013).

The main principle of CT imaging method is to analysis and processing of CT or MRI images (Feinberg et al., 1999) and to extract key features by various reconstruction algorithms to perform three-dimensional reconstruction. The modeling flow chart is shown in Figure 5.

Hollister (2005) performed gray-scale processing of medical images and obtained the distribution of solid voxel and void information using a binarization segmentation algorithm. Then they established the porous structure by mapping the defined porous structure unit to the solid voxel. Podshivalov et al. (2013) segmented the CT image and removed the redundant shadow part, then repaired hole on the model, and finally obtained ideal bone scaffold model, as shown in Figure 4. Ben and Fischer (2015) made important progress in CT image adaptive model reconstruction. They introduced the quadtree and octree algorithms into the process of adaptive model reconstruction, which greatly simplifies the modeling process. Zheng et al. (2020) scanned skull samples, extracted the shape of the skull and reconstructed inside structure of the trabecular. Cell cultures experiment showed that the model restores the internal structure of the skull, and has good biocompatibility. The CT imaging method can produce porous structure closest to the three-dimensional structure inside the bone tissue (Cai, 2008; Cai and Xi, 2009; Cai et al., 2012). However, the method has a high dependence on the image resolution (Li et al., 2006), and the simplification processing of CT or MRI data is relatively cumbersome, which leads to certain restrictions on its clinical application (Jones et al., 2007).

### Comparison of Porous Scaffold Design Methods

At present, CAD design and topology optimization methods are the widely used methods in the design of porous scaffolds because these methods are simple and reliable, and simultaneously meeting the basic requirements of reducing the modulus of the scaffolds. The structures designed by TPMS and Voronoi methods are more similar to the internal structure of human bone, and they have better permeability and mechanical properties than the structure designed by CAD method. The CT imaging method can reflect the real structure of bone. If the reconstruction process of the model can be simplified, it is believed that the CT imaging method can be further developed. The comparisons of these modeling methods are summarized, as shown in Table 4.

**TABLE 4:** Comparison of porous scaffold design methods.

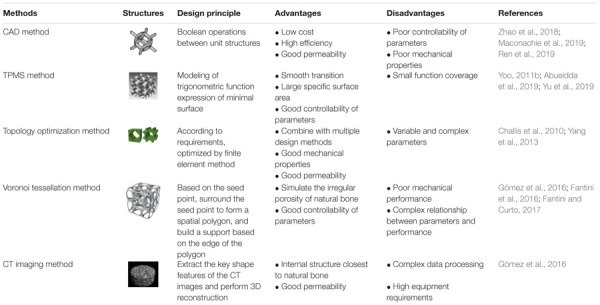

The above is the comparison and summary of structural unit design methods. In addition, we also pay attention to the overall design of the scaffold, and the hierarchical structure is the hot spot in the overall design of the scaffold (He et al., 2021). The real shape of bone in human body is the porous structure with gradient distribution. According to the size and porosity of the pores, bone can be divided into dense and cancellous bone from the outside to the inside (Du et al., 2019). Only vascular and nerve channels remain in dense bone, with a porosity of about 5–30%. Cancellous bone has a porosity of 30–90%, and can deform under stress and absorb energy shocks from outside (Liu et al., 2018). The hierarchical structure of natural bone in human body can not only meet the needs of material transportation, but also meet the requirements of mechanical properties. Singh et al. (2010) and Huang et al. (2014) demonstrated that the hierarchical structure of the scaffold can produce anisotropic mechanical properties, which are more similar to the mechanical properties of human bones than the homogeneous structure. If the unit design is combined with the overall hierarchical design, scaffolds with better comprehensive performance can be obtained.

## Summary and Outlook

Additive manufacturing technology provides unprecedented opportunities for the production of customized biomedical implants. With the development of materials science and computer-assisted technologies, metal porous scaffolds produced by AM, additive manufacturing have been applied in clinical practice. In the future, the preparation of porous metal scaffolds by AM, additive manufacturing still has great potential in the following fields.

(1) The metal scaffolds with degradable materials can effectively reduce the subsequent maintenance problems of the implant. However, the most widely used materials for metal porous scaffolds are still non-degradable metals such as pure Ti, Ti alloys, 316L and so on. So it is particularly important to design and prepare new biodegradable materials that matching degradation rate with bone tissue.
(2) Real bone in the human body has gradient microstructures; thus the development of porous scaffolds with gradient structure is a future development trend. At present, it is challenging to obtain a gradient scaffold with better performance with a single design method. Therefore, combination methods of topology optimization, CAD and minimal surface and so on. Can be tried to design the gradient structure in the future.
(3) Surface modification can effectively improve the osteogenesis, bacteriostasis, and biocompatibility of porous scaffolds. At present, preparation of inorganic and organic surfaces, or changing the surface morphologies of bone implants are the main surface modification methods. In the future, new surface modification materials and methods used for porous scaffolds should be developed in order to improve its biocompatibility or realize the treatment of certain diseases.
(4) At present, most of the researches on the biocompatibility of the scaffold only stays in cell experiments, which lacks accurate evaluation of the scaffold performances. Thus effective *in vivo* osteogenic experiment should be introduced and biological standards should be established to more scientifically evaluate the osteogenic ability of porous scaffolds.
(5) 4D printing is a concept that has emerged in recent years, which generally refers to programmatical change in shape and function of 3D printed scaffolds over time. The change can adjust the mechanical properties or structure characteristics of the porous scaffolds and expand its functions and applications, providing a broader prospect for the development of porous scaffolds.

## Author Contributions

YT carried out the conception of the idea of the manuscript. ZQ and JL provided the data and advice. BW and GL collected and collated the data. YL wrote the original draft. EL reviewed and revised the original draft. KX and CL provided guidance for the revision of the manuscrip. LW provided the financial support for the project to this publication. All the authors contributed to the article and approved the submitted version.

## Conflict of Interest

The authors declare that the research was conducted in the absence of any commercial or financial relationships that could be construed as a potential conflict of interest.
